# Young key affected population in Myanmar: are there any challenges in seeking information and care for HIV/sexually transmitted infections and reproductive health?

**DOI:** 10.12688/f1000research.16029.2

**Published:** 2018-11-13

**Authors:** Kyaw Min Htut, Myo Myo Mon, Zin Mar Aye, Lwin Lwin Ni

**Affiliations:** 1 Department of Medical Research, Ministry of Health and Sports, Yangon, 11191, Myanmar

**Keywords:** Young Key Affected Population, Men who have sex with men, Female sex workers, HIV, Sexually Transmitted Infection, Reproductive Health, Myanmar

## Abstract

**Background:** Unmet needs and barriers in seeking HIV/STI and RH information and care are present especially among young key affected population (YKAP). Therefore, the study was conducted to determine the health seeking behaviors of YKAP regarding HIV/STI and RH, and challenges in seeking health information and care.

**Methods:** A cross-sectional, mixed-methods study was conducted at two large cities in Myanmar. Face-to-face interviews were conducted with YKAP aged 15-24 years. In-depth interviews and key informant interviews were done with YKAP and health care providers. Descriptive statistics and bivariate analyses were done for quantitative data and thematic analysis was applied for qualitative data.

**Results: **A total of 119 young men who have sex with men (YMSM) and 123 young female sex workers (YFSW) included in the study. Mean age of YMSM and YFSW were 20.9±2.4 and 21.7±2.2 years. Over 30% of YMSM and 49.3% of YFSW had experience of any STI symptom. Particularly, 17% of YMSM and 10% of YFSW had genital ulcer, and majority sought health care at NGO clinics. About 37% of YMSM and 40% of YFSW visited Drop-in-center (DIC) within one to six months. Over 13% of YMSM and 14.6% of YFSW had challenges in seeking HIV/STI and RH information.  YMSM/YFSW type and age of YMSM were associated with visit to DIC. Lesser proportions of Tha-nge (43.5%), younger age YMSM (66.7%), brothel-based YFSW (47.9%) visited DIC than others (p<0.05). Challenges and unmet needs expressed by YKAP were reluctance in asking health information, worry for future fertility, consequences of anal sex and contraception. Challenges expressed by providers were limited time during outreach service and difficulty in reaching entertainment-based sex workers.

**Conclusions:** Special attention in provision of health information should be paid to YKAP since there is a considerable proportion of YKAP with unmet need in seeking HIV/STI/RH information and care.

## Introduction

Globally, one-fourth of the total population is young people aged 10–24 years, and they are most vulnerable from the global epidemic of HIV. Around one-third of all new HIV infections worldwide occurred among youth aged 15–24 years and about five million people aged 10–24 years were infected with HIV ^1^. As described in latest census of Myanmar in 2014, the total population is 51.4 million, with 16 million young people, which accounts for 28% of the population ^2^. One of the main health problems faced by young people results from sexual and reproductive health risk-taking behaviors, leading to unintended pregnancies and HIV/AIDS.

Key affected populations in relation to HIV transmission were men who have sex with men (MSM), sex workers, people who inject drugs, and people in prisons ^3^. In Myanmar, according to sentinel surveillance data, HIV prevalence among young key populations was higher than that of other populations. In particular, 5.5% and 7.9% among female sex workers aged 15–19 years and 20–24 years, respectively; and 9.1% and 8.6% among men who have sex with men aged 15–19 years and 20–24 years, respectively ^4^. As mentioned in National Strategic Plan on HIV/AIDS for 2016–2020, HIV prevalence among female sex workers (FSW) and MSM were 14.6% and 11.6% respectively according to findings from the Integrated Biological and Behavioral Survey in 2015 ^5^.

Previous studies have identified factors related to health care-seeking behaviors of young people. These factors included stigma and discrimination ^6^, long waiting times, inconvenient locations of clinics, not knowing where to get the services, and negative attitudes among health care providers ^6, 7^. Globally, there are a number of studies indicating the presence of HIV‑related stigma in healthcare settings ^8, 9^. Discrimination is a major problem when seeking health care for HIV-infected individuals ^10^. Research had found that consequences of HIV-related stigma on health-seeking behavior resulted in fear of receiving HIV testing, and delaying in responses such as adhering to treatment and preventive behaviours ^11, 12^. In a study in Laos, the main barriers were related to location of health facility, lack of awareness on availability of services and unfavorable attitude of healthcare providers ^7^.

With regards the service utilization, the percentage of FSW who received an HIV test in the last 12 months was 71%, and the percentage of MSM who received an HIV test in the last 12 months was 48% ^13^. Reducing the incidence of HIV among priority populations like MSM and FSW was described as one of the objectives to fulfil the goal of the current National Strategic Plan. It was also stated that efforts must be made to tailor services to reach the priority population of young people ^5^. However, very few studies have been conducted among the YKAP in Myanmar identifying health-seeking behaviors and their perceived barriers. Therefore, current study was conducted to determine the health seeking behaviors regarding HIV/STI and reproductive health (RH), challenges and the unmet needs in seeking health information among YKAP.

## Methods

### Study design and setting

A cross-sectional, mixed-methods study was conducted using both quantitative and qualitative methods among the YKAP, including young FSW (YFSW) and young MSM (YMSM) in Yangon and Mandalay, Myanmar, during February and June 2017. Yangon and Mandalay are two largest business cities of Myanmar where the YKAP community is larger than that of other areas.

### Participants

***Inclusion criteria:***

1. YFSW aged 15 to 24 years currently working as sex workers whose sex work was based either at brothels, entertainment places (karaoke, club, bar) or on the streets.
2. YMSM aged 15 to 24 years who identified themselves as apwint (open type) or apone (hidden type) or tha-nge (male partner of either apwint or apone).

Operational definition of MSMs according to their types ^5^:

Apwint: Those who are biological males whose public and private gender identity is generally feminine, but they may dress as men or dress and act as females. Apwint are generally more ‘open’ MSM and some could be considered ‘transgender’.
Apone: Those who are biological males whose gender identity may be either masculine or feminine and may or may not express themselves femininely.
Tha Nge: Those who are biological males whose gender identity is masculine with a sexual preference for apwint and apone as well as for women, however they are often ‘hidden’ MSM.

Operational definitions of RH and unmet needs in seeking health information was defined in our study as follows.

Reproductive health and services

According to World Health Organization, “reproductive health addresses the reproductive processes, functions and system at all stages of life and it implies that people are able to have a responsible, satisfying and safe sex life and that they have the capability to reproduce and the freedom to decide if, when and how often to do so”. In present study, we focused only on STI/HIV and the services related to them.

Unmet needs in seeking health information

“Although YKAP wants to know or receive STI/HIV information/care, they could not get/receive information/care as they would like to.”

For example- though they want to know details about the consequences of anal sex, they do not know how to get or from whom they could get the information.

### Variables

*Outcome variables*

Health-seeking behavior was measured on

1) Ever receive HIV testing (Yes/No)
2) STI treatment (Yes/No)
3) Visit to DIC (Yes/No)

*Independent variables*

1) Age: both continuous and categorical measurement
2) Type of MSM: either apwint, apone or thange (as defined above)
3) Type of FSW: either brothel-based, entertainment-based or street-based
4) Education: either illiterate, read & write, primary school, middle school, high school, university
5) Having income earning job: either yes, not always or no
6) Any STI symptoms: yes or no

Challenges and unmet needs were mainly discussed during in-depth interviews and key informant interviews.

### Sampling and sample size

Purposive sampling was applied in recruiting YMSM and YFSW since they were hidden population and could not be easily accessible like general population. Firstly, identification of the places for recruitment of the possible participants was made after discussion with the focal persons from the networks of FSW and MSM. FSWs were recruited from brothels, massage parlors, karaokes and soliciting sites on the streets according to the inclusion criteria. MSMs were recruited at beauty salons and gathering places along the streets. No recruitment was done through clinics and drop-in centers (DIC) to prevent bias in sampling those with good health-seeking behavior. Participants for qualitative interviews were selected based on the type of YMSM and YFSW, their experience of barriers/unmet needs and their willingness to participate in the interviews.

Considering proportions of MSM and FSW who seek HIV testing service in last 12 months as 20% and 30% according to a previous study ^5^, 95% confidence level, precision of 0.1 and design effect of 1.5, the minimum required sample size for each population were 93 (YMSM) and 122 (YFSW) by using a sample size formula for one proportion.

### Data collection

Research assistants were trained at the Department of Medical Research before field data collection. A structured questionnaire was developed for quantitative assessment and face-to-face interviews were carried out with YKAP using a structured, pre-tested questionnaire by trained interviewers.

Guidelines were developed for in-depth interviews (IDIs) and key informant interviews (KIIs). In-depth interviews were conducted with YKAP and key informant interviews were carried out with the service provider to explore their opinions and experiences. Service providers are the focal persons from National AIDS Program and non-governmental organization (NGO)/international NGO working for the key populations. These IDIs and KIIs were conducted by two principal investigators who have experience of conducting qualitative interviews. Because of confidentiality issues, the interviews were not audio recorded. However, discussions were noted down by well-trained note takers. Two note-takers also have previous experience of dealing with key populations and they were mainly trained on how to strictly ensure and value confidentiality of the study population.

A total of 119 face to face interviews with YMSM and 123 face to face interviews with YFSW were carried out using a structured questionnaire. In addition, 12 IDI and 10 KII were conducted with service providers and focal persons. ( Supplementary File 1, Supplementary File 2), ( Supplementary File 3)

Main themes included in IDIs and KIIs were as follows:

- perspective on young key affected population

- challenges in receiving/providing RH/HIV information and health care

- unmet needs in receiving RH/HIV information and health care

### Data management and analysis

Data entry was conducted using EpiData version 3.1 and analysis was conducted with SPSS version 16 for quantitative data. Descriptive statistics were shown as frequency/percentage for categorical variables and mean/median for continuous variables. Bi-variate analysis was done using the chi-squared test.

Regarding the qualitative data, transcripts were prepared and manual coding was applied to explore the main themes such as unmet needs and barriers in seeking HIV/STI and other RH information. Manual thematic analysis was done by using matrix according to the themes and type of participants.

### Ethical consideration

Verbal informed consent was obtained from each participant after thorough explanation about the objectives of the study. Regarding the participants below 18 years, we have to request their consents directly since it was not possible to ask from their guardians. Anonymity and confidentiality of the information were ensured using the code numbers and only researchers have accessed to the information. Ethics approval was also obtained from the Ethics Review Committee of The Department of Medical Research (Ethics/DMR/2016/091), Ministry of Health and Sports, Myanmar.

## Results

### Participant characteristics

Socio-demographic characteristics and family related information of participants are shown in Table 1. A total of 119 young men who have sex with men (YMSM) and 123 young female sex workers (YFSW) included in the assessment. The mean age of YMSM and YFSW was 20.9±2.4 and 21.7±2.2 years, respectively. Nearly 60% of YMSM were apwint (open type), 21.8% were apone (hidden type) and 19.3% were tha-nge (male partner of apwint or apone) as identified by themselves. Based on the place of sex work, YFSW included in the study were identified as brothel-based (40%), entertainment-based (karaoke/restaurant/nightclub/massage) (32.5%) and street-based (28.5%) respectively.

**Table 1.** Background characteristics and family-related information of the participants.

Characteristics	YMSM (n=119), n (%)	YFSW (n=123), n (%)
**Age, years**		
Mean ± SD	20.9 ± 2.4	21.7 ± 2.2
Range	16 – 24	16 – 24
**Education**		
Illiterate	3 (2.5)	16 (13.0)
Read & write	7 (5.9)	25 (20.3)
Primary school	26 (21.8)	51 (41.5)
Middle school	49 (41.2)	29 (23.6)
High school	10 (8.4)	1 (0.8)
Graduate/University	24 (20.2)	1 (0.8)
**Marital status**		
Married	32 (27.1)	30 (24.4)
Not married	85 (71.2)	56 (45.5)
Divorced	2 (1.7)	37 (30.1)
**Have income earning job**		
Yes, always	87 (73.1)	15 (12.2)
Yes, not regular	18 (15.1)	5 (4.1)
No	14 (11.8)	103 (83.7)
**Monthly income, Kyats**		
No income	12 (10.1)	-
Up to 1000,000	18 (15.1)	6 (4.9)
>100,000 – 200,000	53 (44.5)	46 (37.4)
>200,000 – 500,000	28 (23.5)	53 (43.1)
>500,000	8 (6.7)	18 (14.6)
**Current living conditions**		
Parents/Guardians	76 (53.9)	49 (39.9)
Friends/colleagues	22 (18.4)	66 (53.7)
Partner	13 (1.1)	5 (4.1)
Alone	8 (6.7)	3 (2.4)
**Parents/Guardians** **accepted as MSM/FSW**		
Accepted	67 (56.3)	27 (22.0)
Not accepted	21 (17.6)	17 (13.8)
Don’t know their status	31 (26.1)	79 (64.2)

YMSM, young men who have sex with men; YFSM, young female sex workers.

Regarding their education status, 72.3% and 86.2 % of YMSM and YFSW, respectively, had completed primary school education, and 8.4% of YMSM were university graduates. Around one-fourth of both YMSM (27.1%) and YFSW (24.4%) were married. Median monthly income of YMSM and YFSW were 200,000 Kyats and 300,000 Kyats, respectively. Over 50% of YMSM and about 40% of YFSW were currently living with their parents. More than half of YMSMs’ parents/guardians accepted their sexual identity as MSM while only 22% of YFSWs’ parents/guardians accepted them as sex workers.

### Sexual health of participants

**Figure 1.**
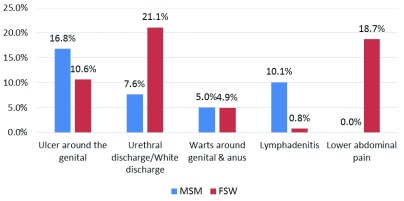
STI symptoms experienced by young men who have sex with men (YMSM) and young female sex workers (YFSW).

Figure 1 describes the STI symptoms experienced by YMSM and YFSW; genital ulcer was most common for YMSM while white discharge was most common for YFSW. In particular, past incidence of genital ulcers was reported by 17% of YMSM and 11% of YFSW. Over 21% of YFSW suffered from white discharge while 7.6% of YMSM suffered from urethral discharge. Additionally, lower abdominal pain was also common in YFSW (18.7%). Table 2 shows the health-seeking behaviors of YMSM and YFSW regarding RH, STI and HIV. About 70% of YMSM and 56% of YFSW had experience of health-seeking for STI symptoms and the majority of them go to NGO clinics to treat STI. Over 90% of YMSM and YFSW have received HIV testing in the past and over 80% of them had tested for HIV within 6 months. The main reason for undergoing HIV testing is that they would like to know whether they have been infected with HIV or not. Over 90% of both YMSM and YFSW went to an NGO clinic for HIV testing. Regarding the utilization of drop-in centers (DICs), 79% of YMSM and 56.9% of YFSW have ever visited a DIC. Among them, 53.2% of YMSM and 38.6% of YFSW visited a DIC within the previous month.

**Table 2.** Health seeking behaviours of young men who have sex with men (YMSM) and young female sex workers (YFSW) regarding reproductive health and STI/HIV services.

Characteristics	YMSM (n=119), n (%)	YFSW (n=123), n (%)
**Ever have any STI symptom**		
Yes	36 (30.3)	54 (49.3)
No	83 (69.7)	69 (56.1)
**Experience of health** **seeking for STI symptoms**		
Yes	26 (72.2)	35 (64.8)
No	10 (27.8)	19 (35.2)
**Place of seeking health care** **for STI**	(n=26)	(n=35)
NGO clinic	22 (84.6)	25 (71.4)
Private clinic/hospital	2 (7.7)	8 (22.9)
Public clinic/hospital	2 (7.7)	2 (5.7)
**Ever received HIV testing**		
Yes	112 (94.1)	112 (91.1)
No	7 (5.9)	11 (8.9)
**Last time of HIV testing** **(n=112)**		
Within 6 months	93 (83.1)	91 (81.3)
6 months–1 year	11 (9.8)	13 (11.6)
>1 year	8 (7.1)	8 (7.1)
**Reason of HIV testing**	(n=112)	(n=112)
Friends also take testing	23 (20.5)	6 (5.4)
NGO staff come & ask for testing	6 (5.4)	40 (35.7)
Want to know my HIV status	75 (67.0)	62 (55.4)
Others	8 (7.6)	4 (3.6)
**Place of HIV testing**	(n=112)	(n=112)
NGO clinic	102 (91.1)	102 (91.1)
Private clinic/hospital	2 (1.8)	7 (6.2)
Public clinic/hospital	8 (7.1)	2 (1.9)
Others	-	1 (0.9)
**Ever visited DIC**		
Yes	94 (79.0)	70 (56.9)
No	25 (21.0)	53 (43.1)
**Last visit to DIC**	(n=94)	(n=70)
Within 1 month	50 (53.2)	27 (38.6)
1–6 months	35 (37.2)	28 (40.0)
6 months–1 year	5 (5.3)	4 (5.7)
>1 year	4 (4.3)	11 (15.7)

NGO, non-governmental organization; DIC, drop-in clinic.

**Figure 2.**
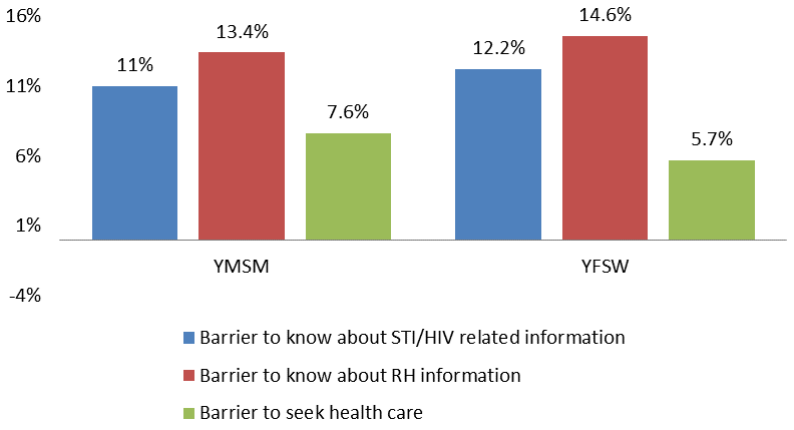
Barriers or limitations in receiving STI/HIV and reproductive health (RH) information, and health care seeking. YMSM, young men who have sex with men; YFSM, young female sex workers.

Figure 2 shows the barriers or limitations in receiving STI/HIV and RH information, and health care seeking. Just over 13% of YMSM and 14.6% of YFSW mentioned that they experience external and personal barriers towards seeking health information on RH. Similarly, 11% of YMSM and 12% of YFSW have barriers in seeking STI/HIV information.

Visit to DICs among YKAP and their background characteristics are shown in Table 3. Type of MSM and age were associated with visiting a DIC among YMSM. A lesser proportion of tha-nge (43.5%) visited a DIC than apwint (85.7%) and apone (92.3%) (p=0.0001). A higher proportion of older YMSM visited DICs in comparing to younger MSM (84.3% vs. 66.7%, p=0.03). Among YFSWs, visiting a DIC was associated with their place of work: a significantly higher proportion of street-based YFSW (77.1%) visited DICs than those who worked in entertainment locations (50%) and brothels (47.9%) (p=0.01).

**Table 3.** Association between background characteristics and visit to drop-in clinics (DICs) among young men who have sex with men (YMSM) and young female sex workers (YFSW).

Characteristics	Visit DIC		p-value
	Yes, n (%)	No, n (%)	
**YMSM (n=119)**			
**Type of MSM**			0.0001
Apwint	60 (85.7)	10 (14.3)	
Apone	24 (92.3)	2 (7.7)	
Tha-Nge	10 (43.5)	13 (56.5)	
**Age group**			0.03
15–19 years	24 (66.7)	12 (33.3)	
20–24 years	70 (84.3)	13 (15.7)	
**YFSW (n=123)**			
**Type of YFSW**			0.01
Brothel-based	23 (47.9)	25 (52.1)	
Entertainment based	20 (50.0)	20 (50.0)	
Street-based	27 (77.1)	8 (22.9)	
**Age group**			0.14
15–19 years	11 (44.0)	14 (56.0)	
20–24 years	59 (60.2)	39 (39.8)	

During in-depth interviews and key informant interviews, different challenges and unmet needs in seeking health information and services were mentioned by YKAP as shown in Table (4). Common challenges mentioned by YMSMs were “financial problems” and “discrimination from health care providers”, while YFSWs stated their challenges as “no/limited time to access health service”, “reluctance in asking health information” and “restriction to go outside”. Regarding their unmet needs, most tha-nge (male partners of apwint and apone) expressed their concerns about the health consequences from having sexual relationship with MSM and future fertility. Other MSMs would like to know the consequences of anal sex and its treatment. Similarly, YFSWs also expressed that they have unmet needs concerning their future fertility and contraception. Most providers mentioned that it was difficult to reach and provide services to the girls from entertainment locations, such as karaoke.

**Table 4.** Challenges and unmet needs regarding STI/HIV and reproductive health information and care.

Theme	YMSM	YFSW	Provider
**Challenges**	Financial problem	No or limited time to access health service	Difficult to reach entertainment-based sex workers
	Discrimination from health care provider	Reluctance in asking health information	Limited time to provide health messages during mobile service
	Difficulty in accessing health care services due to long distance	Restriction to go outside	Little interest to health messages and
	Reluctance in asking health information		
**Unmet needs**	Health consequences from having sexual relationship with MSM	Future fertility	
	Future fertility	Contraception	
	Consequences of anal sex and treatment		

YMSM, young men who have sex with men; YFSM, young female sex workers.

Selected quotations included:

“… Currently, I’m living together with “achout” (apwint MSM), but I have a plan to marry a girl in the future. I worry about my fertility status at that time… afraid that I may not able to procreate …” (IDI 3, tha-nge, 23 years old).
“… We can’t go outside everyday… we’re allowed to go outside for one day per week … only for 2–3 hours …” (IDI 6, FSW, 20 years old).
“… We could educate the girls from brothel … They told us their experiences frankly… But, we couldn’t communicate frankly with the girls from karaoke … most of them didn’t accept …” (KII 2, out-reach health care provider).

Complete answers to questionnaires for young men who have sex with men and young female sex workers A key to the coding and abbreviations is also included. Click here for additional data file. Copyright: © 2018 Htut KM et al. 2018 Data associated with the article are available under the terms of the Creative Commons Zero "No rights reserved" data waiver (CC0 1.0 Public domain dedication).

## Discussions and conclusions

The current study highlights the health-seeking behaviors, challenges and unmet needs of the YKAP (YMSM and YFSW) regarding reproductive health and STI/HIV services. Experience of any STI symptom was disclosed by some YMSM and YFSW. The majority of YMSM and YFSW have received HIV testing in the past. In addition, both YMSM and YFSW had experience of health-seeking for STI symptoms and majority of them sought health care from NGO clinics. We also found barriers and unmet needs in seeking health information on RH and STI/HIV among YKAP.

Previous studies have documented on the health care seeking behavior among key population like MSM and FSW. Studies conducted in China found that 40–60% of MSM had ever done HIV testing ^14– 16^, while a similar HIV testing rate among FSW was documented in a study done in Nigeria ^17^. Moreover, Bartelsman *et al*. documented that the HIV testing rate was as low as 32.7% among MSM in Amsterdam ^18^. In contrast to other studies, much higher proportions of YMSM and YFSW from current study had been tested for HIV in the past. Different socio-economic background, sampling strategy and cultural context might contribute to this discrepancy. However, findings on high HIV testing rates among YMSM was consistent with the previous study done in two large cities of Myanmar, Yangon and Monywa, in 2015 ^11^. It was also supported by the evidence from the progress report of National AIDs Program in Myanmar, which found that HIV testing rates among MSM and FSW were dramatically increased between 2006 and 2010. Specifically, testing rates tripled in MSM and quadrupled in FSW ^19^. In the current study, although the self-reported testing rates were high, we did not ask about the quality of testing services and did not verify these rates by other methods.

Experience of STIs among FSW had been noted in previous studies ^20, 21^. In Bangladesh, 41.6% of FSW had experience of any STI symptom and many of them had unmet needs for SRH care ^21^. A lesser proportion of YFSW from our study also had experience of STI symptoms in the past. On the other hand, 21.4% and 15.4% of MSM from Tanzania and Peru had any past STI symptoms, and similar findings were also identified in the current study ^22, 23^. However, in our study, experience of STI symptoms was noted according to their responses and was not validated by blood test. Access to RH information, including that concerning HIV/STI, is important for the YKAP.

Unmet needs and the barriers in seeking sexual and reproductive health care was documented in previous studies ^24, 25^. The prevalence of unmet need was 25% among hotel-based FSW and 36% among street-based FSW according to a study in Bangladesh ^25^. Another study also reported that over 50% of FSWs have faced barriers in seeking SRH care ^24^. Common barriers included financial problems, shame about receiving care, unwillingness and unfriendly behavior of the provider. Certain proportions of YKAP from current study mentioned that they have challenges in seeking reproductive health information. Similarly, some of them have barriers in seeking STI/HIV information. One of the main barriers YFSW mentioned was “no or limited time to access health services”. To overcome this barrier, we may need to discuss more with the gate keepers like managers, brothel owners to allow them in receiving mobile health care services or DIC services. Reluctance in asking health information was also expressed by YMSM. In this case, health care providers should have to do more counseling to YKAP.

The current study has certain limitations. Findings on the information related to STI experience and HIV testing of key population may have some bias since we have to rely on the respondents’ answers and could not validate them by other methods. However, we tried to overcome the limitation by providing a thorough explanation about the study’s objectives. Furthermore, generalization of the study findings to other areas of Myanmar may also have limitations because the study participants were only from two large major cities, where many NGOs/international NGOs are working for these populations.

In conclusion, some YKAP have experienced of STI symptom in the past and many of them went to NGO clinic for seeking care. Moreover, many YKAP have tested for HIV within six months. Lesser proportions of Tha-Nge, younger MSM, brothel and entertainment-based YFSW visited DIC than their counterparts. A considerable proportion of YKAP perceived that they have unmet needs in seeking RH information and care.

Special attention in provision of health information should be paid to the YKAP since there is a considerable proportion of the YKAP with unmet needs in seeking RH information and care. Strengthening of health education activities regarding STI is recommended for YKAP, especially for YFSW who work in entertainment-based locations.

## Data availability

The data referenced by this article are under copyright with the following copyright statement: Copyright: © 2018 Htut KM et al.

Data associated with the article are available under the terms of the Creative Commons Zero "No rights reserved" data waiver (CC0 1.0 Public domain dedication). 

**Dataset 1. Complete answers to questionnaires for young men who have sex with men and young female sex workers.** A key to the coding and abbreviations is also included. DOI: https://doi.org/10.5256/f1000research.16029.d217070 ^26^.

Transcripts from interviews are not available to maintain the confidentiality of the study subjects.

## Supplementary material

Supplementary File 1. Questionnaire used to obtain data for young men who have sex with men.

Supplementary File 2. Questionnaire used to obtain data for young female sex workers.

Supplementary File 3. Guidelines for interviews with service providers and the young key affected population.
