# Supplementary material for: Young key affected population in Myanmar: are there any challenges in seeking information and care for HIV/sexually transmitted infections and reproductive health?
Source: F1000Res. 2018 Nov 13;7:1515. Originally published 2018 Sep 21. [Version 2] doi: 10.12688/f1000research.16029.2 (PMC6249637; doi:10.12688/f1000research.16029.2)
Supplement: Supplementary file 3 [file f1000research-7-18428-s0002.tgz › 1e26280c-6362-4db5-a470-09ee07d53f0d_YKAP_FSW_Questionnaire_English.docx]

**Health seeking behaviors of young key affected population regarding reproductive health and STI/HIV services (For YFSW)**

Questionnaire

| Township | (1) Yangon  (2) Mandalay | \|___\| |
| --- | --- | --- |
| Respondent ID |  | \|___\|___\|___\| |
| Date of interview |  |  |

**Section 1 Background characteristics**

| 1.1 | Respondent’s age (completed year) | ________________years | \|___\|___\| |
| --- | --- | --- | --- |
| 1.2 | Current Schooling | (1) Never schooling  (2) School dropout  (3) Graduated  (4) Current schooling | \|___\| |
| 1.3 | Education | (1) Illiterate  (2) Read & write  (3) Primary school  (4) Middle school  (5) High school  (6) Graduate/University | \|___\| |
| 1.4 | Parents’ condition | (1) Both alive  (2) Maternal orphan  (3) Paternal orphan  (4) Double orphan  (5) Others (specify) _________ | \|___\| |
| 1.5 | Do you have any job which could earn income apart from sex work? | 1. Yes, always 2. Yes, not regular 3. No (Skip to Q:1.7) | \|___\| |
| 1.6 | If yes, occupation of the respondent | 1. Manual/unskilled labour 2. Private/Government employee 3. NGO/INGO staff 4. Own business 5. Others (specify) ___________ | \|___\| |
| 1.7 | Estimated monthly income (Kyats) | __________________ | \|___\|___\|___\|___\|___\|___\|___\| |
| 1.8 | Current living condition | 1. Live with parents 2. Live with either parent 3. Live with friends 4. People from work place 5. Others (specify) ___________ | \|___\| |
| 1.9 | Do your parents or guardians accept you are working as sex worker? | 1. Yes 2. No 3. Don’t know | \|___\| |
| 1.10 | Marital status | 1. Married 2. Not married 3. Divorced | \|___\| |

| 1.11 | Type of FSW | 1. Brothel house 2. Entertainment based (Karaoke/Club) 3. Street-based 4. Others (specify) ___________ | \|___\| |
| --- | --- | --- | --- |

**Section 2 STI and HIV related health seeking behaviours**

| 2.1 | Have you ever suffered from these symptoms?  (Multiple response) | (1) Ulcer around the genital  (2) White discharge  (3) Warts around the genital and anus  (4) Inguinal lymph node enlargement  (5) Lower abdominal pain  (6) Never suffered | \|___\|  \|___\|  \|___\|  \|___\|  \|___\|  \|___\| |
| --- | --- | --- | --- |
| 2.2 | If yes, did you seek health care? | (1) Yes  (2) No | \|___\| |
| 2.3 | Where did you go to seek the healthcare? | 1. NGO clinic 2. Private clinic/hospital 3. Public clinic/hospital 4. Others (specify)__________ | \|___\|  \|___\|  \|___\|  \|___\| |
| 2.4 | Have you ever received HIV testing? | 1. Yes 2. No | \|___\| |
| 2.5 | If yes, how many times? | __________ | \|___\|___\| |
| 2.6 | When was the last time? | 1. Within 2 weeks 2. Within 2 weeks to 1 month 3. Between 1 – 6 month 4. Between 6 month – 1 year 5. > one year | \|___\| |
| 2.7 | Why did you take the test? | 1. Because friends also take test 2. Needs to test for applying job 3. NGO staff comes & ask for test 4. Others (specify) | \|___\|  \|___\|  \|___\|  \|___\| |
| 2.8 | Where did you take the test? | (1) NGO clinic  (2) Private clinic/hospital  (3) Public clinic/hospital  (4) Others (specify) __________ | \|___\|  \|___\|  \|___\|  \|___\| |
| 2.9 | Do you have any barrier or limitation to know about STI/HIV related information? | (1) Yes  (2) No | \|___\| |
| 2.10 | Do you have any barrier or limitation to know about reproductive health related information? | (1) Yes  (2) No | \|___\| |
| 2.11 | Do you have any barrier or limitation when you need to seek health care? | (1) Yes  (2) No | \|___\| |
| 2.12 | Have you ever visit DIC for FSW run by NGO/INGO | (1) Yes  (2) No | \|___\| |
| 2.13 | If yes, how often | __________weeks | \|___\|___\| |
