# Supplementary material for: Young key affected population in Myanmar: are there any challenges in seeking information and care for HIV/sexually transmitted infections and reproductive health?
Source: F1000Res. 2018 Nov 13;7:1515. Originally published 2018 Sep 21. [Version 2] doi: 10.12688/f1000research.16029.2 (PMC6249637; doi:10.12688/f1000research.16029.2)
Supplement: Supplementary file 4 [file f1000research-7-18428-s0003.tgz › cf078e56-5412-406b-8a28-8f7075f9403b_Provider_guideline.docx]

**Key informant interview (KII) Guideline for Service Providers**

- Introduction about the objectives of the study
- Recording background information of the participant
- Name, Age, Sex
- Designation, Service years

**Theme 1 Young key affected population**

- 1. What do you understand about young key affected population? Why does the term key population refer to? (Probe: MSM, FSW)
  2. Do you think that they have awareness on reproductive health and HIV/STI?
  3. How do you think health seeking behaviors of them regarding reproductive health and HIV/STI service?
  4. Do they come to health facilities when they want to get health care services? What are the conditions they usually asked for health care?

**Theme 2 Challenges in providing health care**

2.1 Do you have any experience of providing health care to them? What about the relationship between service providers and YKAP?

2.2 Are they willing to disclose about their illness to health care providers?

2.3 How do you think about their compliance regarding the treatment?

**Theme 3 Providers’ perspective towards young key affected population**

3.1 Could you kindly tell us your perspective towards them?

3.2 As you already known that they are experienced stigma and discrimination, may I know your perception towards such kind of stigma and discrimination?

3.3 Do you want to provide your services for them when they are seeking health care services?

**Theme 4 Suggestions and recommendations**

4.1 Could you give any suggestion or recommendation concerning health seeking behaviors of them?

**In-depth interview (IDI) Guideline for YKAP**

- Introduction about the objectives of the study
- Recording background information of the participant
- Name, Age, Sex, Education

**Theme 1 Health seeking behaviors regarding HIV/STI and RH among Young key affected population**

- 1. Have you ever experienced of testing for HIV and STI. Could you kindly express me your experience in detail?
  2. How do you think of RH and could you tell me about RH? How do you get the services regarding RH and where?
  3. What about the relationship between service providers and YKAP?
  4. How do you think of providers’ perspective towards YKAP? Could you kindly tell me your experience?
  5. Do YKAP go and seek care at health facilities when they want to get health care services? What are the conditions YKAP usually asked for health care?

**Theme 2 Challenges, barriers and unmet needs**

2.1 Do you have any experience of challenges and barriers on receiving health care?

2.2 Do you have any unmet needs regarding information and services of HIV/STI and RH?
