# Supplementary material for: Young key affected population in Myanmar: are there any challenges in seeking information and care for HIV/sexually transmitted infections and reproductive health?
Source: F1000Res. 2018 Nov 13;7:1515. Originally published 2018 Sep 21. [Version 2] doi: 10.12688/f1000research.16029.2 (PMC6249637; doi:10.12688/f1000research.16029.2)
Supplement: Complete answers to questionnaires for young men who have sex with men and young female sex workers — A key to the coding and abbreviations is also included. [file f1000research-7-18428-s0000.tgz › 85e76596-319e-4a0b-ba3f-3ad3a10e4632_Data.zip!/Data/Guidelines for coding.docx]

**Guideline for YMSM data set**

| **Variable name** | **Variable Description** | **Coding** |
| --- | --- | --- |
| tsp | Township (City) | (1) Yangon  (2) Mandalay |
| age | Respondent’s age in years |  |
| shol | Schooling | (1) Never schooling  (2) School dropout  (3) Graduated  (4) Current schooling |
| edu | Education status | (1) Illiterate  (2) Read & write  (3) Primary school  (4) Middle school  (5) High school  (6) Graduate/University |
| parcon | Parental condition | (1) Both alive  (2) Maternal orphan  (3) Paternal orphan  (4) Double orphan |
| job | Having regular income job | (1) Yes, always (2) Yes, not regular (3) No |
| occu | Occupation | (1) Manual/unskilled labour (2) Private/Government employee (3) NGO/INGO staff (4) Own business (5) Others (specify) _________ |
| inc | Estimated monthly income in Myanmar currency (Kyats) |  |
| livcon | Current living condition | (1) Live with parents (2) Live with either parent (3) Live with friends (4) Live with colleagues (5) Live with partner (6) Stay alone |
| paccept | Parental acceptance as MSM | (1) Yes, accepted (2) No, not accepted (3) Don’t know their status |
| married | Marital status | (1) Married (2) Not married (3) Divorced |
| type | Type of MSM (as perceived by himself) | (1) Ah-pwint (2) Ah-pone (3) Thu-nge (4) Others (specify) _________ |

| sym1 | STI symptom 1  Ulcer around the genital | (1) Yes  (2) No |
| --- | --- | --- |
| sym2 | STI symptom 2  Urethral discharge | (1) Yes  (2) No |
| sym3 | STI symptom 3  Warts around the genital and anus | (1) Yes  (2) No |
| sym4 | STI symptom 4  Lymphadenitis | (1) Yes  (2) No |
| sym5 | History of ever suffered from any STI symptom | (1) Never suffered  (2) Suffered |
| seek | Seeking health care for STI | (1) Yes  (2) No |
| plcseek | Place of seeking care | (1) NGO clinic (2) Private clinic/hospital (3) Public clinic/hospital   Others (specify)__________ |
| hivtest | Ever receive HIV testing | (1) Yes (2) No |
| times | How many times of HIV testing |  |
| last | Last time HIV testing | (1) Within 2 weeks (2) Within 2 weeks to 1 month (3) Between 1 – 6 month (4) Between 6 month – 1 year (5) > one year |
| restest | Reason for HIV testing | (1) Because friends also take test (2) Needs to test for applying job (3) NGO staff comes & ask for test (4) Others (specify) |
| plctest | Place of HIV testing | (1) NGO clinic  (2) Private clinic/hospital  (3) Public clinic/hospital  (4) Others (specify) __________ |
| hivbar | Any barrier in seeking HIV/STI information | (1) Yes  (2) No |
| rhbar | Any barrier in seeking RH information | (1) Yes  (2) No |
| seekbar | Any barrier in seeking health care | (1) Yes  (2) No |
| dic | Ever visit DIC for MSM run by NGO/INGO | (1) Yes  (2) No |
| freq2 | Visit how often | ------ week |
| age_gp | Age group | (1) 15-19 years  (2) 20-24 years |

**Guide for YFSW data set**

| **Variable name** | **Variable description** | **Coding** |
| --- | --- | --- |
| tsp | Township (City) | (1) Yangon  (2) Mandalay |
| age | Respondent’s age in years |  |
| shol | Schooling | (1) Never schooling  (2) School dropout  (3) Graduated  (4) Current schooling |
| edu | Education status | (1) Illiterate  (2) Read & write  (3) Primary school  (4) Middle school  (5) High school  (6) Graduate/University |
| parcon | Parental condition | (1) Both alive  (2) Maternal orphan  (3) Paternal orphan  (4) Double orphan |
| job | Having regular income job | (1) Yes, always (2) Yes, not regular (3) No |
| occu | Occupation | (1) Manual/unskilled labour (2) Private/Government employee (3) NGO/INGO staff (4) Own business (5) Others (specify) _________ |
| inc | Estimated monthly income in Myanmar currency (Kyats) |  |
| livcon | Current living condition | (1) Live with parents (2) Live with either parent (3) Live with friends (4) Live with colleagues (workplace) (5) Live with partner (6) Stay alone |
| paccept | Parental acceptance as MSM | (1) Yes, accepted (2) No, not accepted (3) Don’t know their status |
| married | Marital status | (1) Married (2) Not married (3) Divorced |
| type | Type of FSW (as perceived by himself) | (1) Brothel house (2) Entertainment based (Karaoke/Club) (3) Street-based (4) Others (specify) ___________ |
| sym1 | STI symptom 1  Ulcer around the genital | (1) Yes  (2) No |
| sym2 | STI symptom 2  White discharge | (1) Yes  (2) No |
| sym3 | STI symptom 3  Warts around the genital and anus | (1) Yes  (2) No |
| sym4 | STI symptom 4  Inguinal lymphnode enlargement | (1) Yes  (2) No |
| sym5 | STI symptom 5  Lower abdominal pain | (1) Yes  (2) No |
| sym6 | History of ever suffered from any STI symptom | (1) Never suffered  (2) Suffered |
| seek | Seeking health care for STI | (1) Yes  (2) No |
| plcseek | Place of seeking care | (1) NGO clinic (2) Private clinic/hospital (3) Public clinic/hospital (4) Others (specify)__________ |
| hivtest | Ever receive HIV testing | (3) Yes (4) No |
| times | How many times of HIV testing |  |
| last | Last time HIV testing | (1) Within 2 weeks (2) Within 2 weeks to 1 month (3) Between 1 – 6 month (4) Between 6 month – 1 year (5) > one year |
| restest | Reason for HIV testing | (1) Because friends also take test (2) Needs to test for applying job (3) NGO staff comes & ask for test (4) Others (specify) |
| plctest | Place of HIV testing | (1) NGO clinic  (2) Private clinic/hospital  (3) Public clinic/hospital  (4) Others (specify) __________ |
| hivbar | Any barrier in seeking HIV/STI information | (1) Yes  (2) No |
| rhbar | Any barrier in seeking RH information | (1) Yes  (2) No |
| seekbar | Any barrier in seeking health care | (1) Yes  (2) No |
| dic | Ever visit DIC for MSM run by NGO/INGO | (1) Yes  (2) No |
| freq2 | Visit how often | ------ week |
| age_gp | Age group | (1) 15-19 years  (2) 20-24 years |
