# Supplementary material for: Young key affected population in Myanmar: are there any challenges in seeking information and care for HIV/sexually transmitted infections and reproductive health?
Source: F1000Res. 2018 Nov 13;7:1515. Originally published 2018 Sep 21. [Version 2] doi: 10.12688/f1000research.16029.2 (PMC6249637; doi:10.12688/f1000research.16029.2)
Supplement: Peer review history

# Peer review: Referee response for version 2

**DOI:** 10.5256/f1000research.18428.r40596

**Published:** 2018-11-21

**Authors:** Tasnuva Wahed

**Affiliations:** 1 College of Public Health Sciences, Chulalongkorn University, Bangkok, Thailand

The abstract of revised article still contained some short forms, such as-RH, STIs without full abbreviation. The authors can write as - reproductive health (RH) when they cite it first (please, see first sentence, background of abstract).

 There are no more comments.

I have read this submission. I believe that I have an appropriate level of expertise to confirm that it is of an acceptable scientific standard.

# Peer review: Referee response for version 2

**DOI:** 10.5256/f1000research.18428.r40597

**Published:** 2018-11-16

**Authors:** Kyu Kyu Than

**Affiliations:** 1 Burnet Institute, Melbourne, VIC, Australia

I have no further comments to make.The Authors has revised the article according to my comments and suggestions. I am satisfied with their revision.

I have read this submission. I believe that I have an appropriate level of expertise to confirm that it is of an acceptable scientific standard.

# Peer review: Referee response for version 1

**DOI:** 10.5256/f1000research.17506.r38899

**Published:** 2018-10-16

**Authors:** Kyu Kyu Than

**Affiliations:** 1 Burnet Institute, Melbourne, VIC, Australia

An interesting and important article related to young key population (YKP) in Myanmar. The article mainly focused on the health seeking behaviours of YKPs (Young men having sex with men and Young female sex workers) in relation to STI treatment and HIV testing. It also seeks the utilization of Drop in centres by YKPs. Following clarifications and amendments are need on the manuscript.

 General observation

- Although RH services were included in the objectives, the authors mainly focused on the STI symptoms, HIV testing and DIC utilization.
- The qualitative part of the study was weak in analysis and did not identify the main themes analysed. Was not clear what themes were ask and analysed and what was the added value to the quantitative study.
- Methodology needs to be more elaborated for the mix method studies using specific guidelines for qualitative and quantitative research methods.
- Clearly defining the unmet need for health information seeking is required.

Specific observation

 Abstract

- Sample size for the qualitative and quantitative study should be moved to the methodology paragraph.
- Abbreviations should be avoided if possible in the abstract. If need to please specify before using it.

Introduction

 As the focus of the study is youth 15 to 24 years, the first paragraph is a bit confusing shifting from 10-24 and 15-24 in the same sentence.

 Methods

- There are three main outcome variables that seem to measure the health seeking behaviour. There is a missing variable on obtaining health information. Please clarify.
- Rationale for use of purposive sampling needs to be specified and more details of the sampling and data collection procedures are required. Recruitment of participants for the qualitative study was not mentioned and the sample size for KIIs and IDIs was not observed. Using note takers in the interviews for populations like MSMs and FSWs,how was the breach of confidentiality ensured. 
- Advantage of using a mix method study would benefit the study methodology stronger.
- Need to specify the main themes analysed for qualitative thematic analysis.

Results

- There is lack of results on health information seeking. Not clear how many percent try to seek health information and what are the main barriers? Table 4 describes the qualitative analysis but the linkage is missing with the quantitative findings.
- Description of the qualitative findings seems more appropriate if it could be linked to the quantitative data. Very little qualitative information is observed.

Discussion

- Discussions need a conclusion paragraph to draw the main findings and a way forward.

I have read this submission. I believe that I have an appropriate level of expertise to confirm that it is of an acceptable scientific standard, however I have significant reservations, as outlined above.

# Response

**Published:** 2018-11-01

**Authors:** Myo Myo Mon

**Affiliations:** Department of Medical Research, Ministry of Health and Sports, Myanmar

General Observation

Comment: Although RH services were included in the objectives, the authors mainly focused on the STI symptoms, HIV testing and DIC utilization.

Response: Thanks so much for your comments and clarification. You are right that there are many issues under the scope of RH including pregnancy, child birth, abortion, etc. However, in our study, we would like to focus only on STI and HIV since these are most common problems among MSMs and FSWs in Myanmar especially in relation to HIV transmission.

Comment: The qualitative part of the study was weak in analysis and did not identify the main themes analysed. Was not clear what themes were ask and analysed and what was the added value to the quantitative study.

Response: Thanks for your comments. Our objective for qualitative inquiry is to know their challenges and barriers in seeking health information on HIV/STI. It's aimed to supplement the quantitative information by asking their reasons and perception. Now, we've revised in our manuscript adding the themes we've discussed.

Comment: Methodology needs to be more elaborated for the mix method studies using specific guidelines for qualitative and quantitative research methods.

Response: We've now added more information in the methodology section.

Comment: Clearly defining the unmet need for health information seeking is required.

Response: Now, we had added the operational definition in the manuscript.

Specific observation

Abstract

Comment: Sample size for the qualitative and quantitative study should be moved to the methodology paragraph.

Response: We've already revised in current version.

Comment: Abbreviations should be avoided if possible in the abstract. If need to please specify before using it.

Response: Already revised.

Methods:

Comment: There are three main outcome variables that seem to measure the health seeking behaviour. There is a missing variable on obtaining health information. Please clarify.

Response: We've added the information in current version.

Comment: Rationale for use of purposive sampling needs to be specified and more details of the sampling and data collection procedures are required. Recruitment of participants for the qualitative study was not mentioned and the sample size for KIIs and IDIs was not observed. Using note takers in the interviews for populations like MSMs and FSWs, how was the breach of confidentiality ensured.

Response: Regarding rationale of purposive sampling and sample size for KII & IDI were added in current version of the manuscript. For using note takers in the interviews, we tried our best to ensure confidentiality. Our two note takers are well trained and have experienced in dealing with key population. Aim and objectives of the study were also thoroughly explained to YKAP.

Comment: Need to specify the main themes analysed for qualitative thematic analysis.

Response: we've revised it.

Results:

Comment: There is lack of results on health information seeking. Not clear how many percent try to seek health information and what are the main barriers? Table 4 describes the qualitative analysis but the linkage is missing with the quantitative findings.

Response: In table 2, we've described health seeking for STI, HIV testing and visit to DIC. In this regards, visit to DIC was a proxy measure for health information seeking. Health information seeking was also discussed during the qualitative interview.

Discussion:

Comment: Discussions need a conclusion paragraph to draw the main findings and a way forward.

Response: We've added a conclusion paragraph in current version.

# Peer review: Referee response for version 1

**DOI:** 10.5256/f1000research.17506.r38597

**Published:** 2018-10-08

**Authors:** Tasnuva Wahed

**Affiliations:** 1 College of Public Health Sciences, Chulalongkorn University, Bangkok, Thailand

**Comments:**

 This is an important article in the field of sexual and reproductive health research, especially on sexually transmitted infections (STIs) and AIDs. This study intended to document the health seeking behaviours including barriers in health seeking and access to SRH information among young key populations, eg., female sex workers (FSWs), men having sex with men (MSMs) who were 15 to 24 years old in two cities of Myanmar. Following clarifications are needed on this script:

**General observations:**

- Need a clear operational definition of reproductive health (RH) and services? The author only described about STI and HIV. There are other RH problems of FSWs, such as-unintended pregnancies or childbirth, abortion etc. Did the authors exclude these RH problems from their study?
- Need a clear definition of unmet needs in seeking health information. How did they calculate unmet need of seeking health information?
- Need separately discuss about qualitative and quantitative data collection methods in the methodology.
- Need one paragraph conclusion at the end of this script. At current state, it is finished with limitations following recommendations without a conclusion.

**Specific observations:**

***Abstract: ***

- Background: Please, use full abbreviation at first once before you use short term like sexually transmitted infections (STIs), reproductive health (RH) etc.
- Methods: What is the study period? How many face to face interviews, in-depth interviews and key informant interviews were conducted? What is the main outcome variable?

***Introduction:***

- What is the proportion of **young** key infected populations in Myanmar? If this information is not available, what proportion key infected populations of total population were reported in most recent national census or survey?

***Methods:***

- Operational definition: As mentioned earlier, please, define RH seeking behaviour
- Variables: You have only three outcome variables which are 1) ever receive HIV testing 2) STI treatment 3) Visit to DIC. But you have an objective to determine in seeking health information, therefore, you should have one variable “access to SRH information or seeking health information”.
- Sampling: What is the reason of choosing purposive sampling?
- How sample size of qualitative interviews (eg., in-depth interviews and key informant interviews) were determined?
- As mention earlier, please describe separately and clearly each method of data collection including sampling, sample size, development of questionnaires/ qualitative guidelines, data collection procedure
- Data analysis: Please, describe details of qualitative data analysis.
- Ethical consideration: Were verbal or written informed consents taken? What measures were taken to take consents from participants who were aged below 18 years old?

***Results:***

- You just showed reported barriers in seeking STI/HIV information in Figure 2. Before that, did you measure what proportion of participants accessed to STI/HIV information?
- Table 4: “No or limited time to access health services”- this statement is not clear. Does it mean that YFSW do not have time or have limited time to access health services? If it is so, do you discuss this point how policy makers or health programme can overcome this problem?
- Table 4: “Reluctance in asking health information”- same comment above that this statement is not clear. Does it mean that YFSW kept reluctance in asking health information to healthcare providers? If it is so, do you discuss this point how policy makers or health programme can overcome this problem?

***Discussion:***

- Last sentence of 2 ^nd^ paragraph: “In the current study, although the self-reported testing rates were high, we did not ask about the quality of testing services and did not verify these rates by other methods.”- do you please, justify why you did not take measures on quality and verification of self-reported testing services? How this limitation can be overcome in your study?
- Please, include a concluding paragraph.

I have read this submission. I believe that I have an appropriate level of expertise to confirm that it is of an acceptable scientific standard, however I have significant reservations, as outlined above.

# Response

**Published:** 2018-11-01

**Authors:** Myo Myo Mon

**Affiliations:** Department of Medical Research, Ministry of Health and Sports, Myanmar

Comment: Need a clear operational definition of reproductive health (RH) and services? The author only described about STI and HIV. There are other RH problems of FSWs, such as-unintended pregnancies or childbirth, abortion etc. Did the authors exclude these RH problems from their study?

 Response: Thanks so much for your comments and clarification. You are right that there are many issues under the scope of RH including pregnancy, child birth, abortion, etc.

 However, in our study, we would like to focus only on STI and HIV since these are most common problems among FSWs community in Myanmar especially in relation to HIV transmission. We had revised our manuscript by adding operational definition of RH and services.

 We also revised the following facts according to your comment "Need a clear definition of unmet needs in seeking health information. How did they calculate unmet need of seeking health information?"

 Response: Since our main focus was on STI and HIV, participants were requested to respond about their unmet needs in seeking health information regarding STI/HIV.

 Operational definition of unmet needs in seeking health information was defined in our study as follows.

 “Although YKAP wants to know or receive STI/HIV information/care, they could not get/receive information/care as they would like to.”

 For example- though they want to know details about the consequences of anal sex, they do not know how to get or from whom they could get the information.

 Comment: Need separately discuss about qualitative and quantitative data collection methods in the methodology.

 Response: According to your suggestion, we revised our manuscript by describing separately about quantitative and qualitative methods.

 Comment: Need one paragraph conclusion at the end of this script. At current state, it is finished with limitations following recommendations without a conclusion.

 Response: I think, conclusion is already included together with recommendation. However, we’re revising by adding conclusion statement.

 “In conclusion, some YKAP have experienced of STI symptom in the past and many of them went to NGO clinic for seeking care. Moreover, many YKAP have tested for HIV within six months. Lesser proportions of Tha-Nge, younger MSM, brothel and entertainment-based YFSW visited DIC than their counterparts. A considerable proportion of YKAP perceived that they have unmet needs in seeking RH information and care.”
